# Novel actionable *ROS1::GIT2* fusion in non-Langerhans cell histiocytosis with central nervous system involvement

**DOI:** 10.1007/s00401-022-02520-6

**Published:** 2022-11-23

**Authors:** Gábor Bedics, Monika Csóka, Lilla Reiniger, Edit Varga, Zoltán Liptai, Gergő Papp, Anna Bekő, Catherine Cervi, Csaba Bödör, Bálint Scheich

**Affiliations:** 1 Department of Pathology and Experimental Cancer Research, Semmelweis University, Üllői út 26., Budapest, 1085 Hungary; 2 HCEMM-SU Molecular Oncohematology Research Group, Department of Pathology and Experimental Cancer Research, Semmelweis University, Üllői út 26., Budapest, 1085 Hungary; 3 2nd Department of Pediatrics, Semmelweis University, Tűzoltó u. 7-9., Budapest, 1094 Hungary

The group of histiocytic neoplasms affecting the central nervous system (CNS) includes rare entities with diverse clinicopathological features and variable prognosis. The diagnosis is frequently challenging due to the overlapping histopathological and molecular features [6]. Besides classical oncological treatment modalities, molecularly targeted therapies have been shown to be highly efficacious in this disease group. The most common actionable alterations include *BRAF* p.V600E mutation and other variants affecting the MAPK pathway [4]. The most recently described, predominantly pediatric ALK-positive histiocytosis [3] sometimes presents with isolated CNS involvement [10]. Due to the invariable presence of *ALK* fusions in these tumors, ALK inhibitors have been shown to exert durable effect [9]. Here, we report a pediatric case of non-Langerhans cell histiocytosis affecting the CNS and harboring a novel *ROS1* fusion (*ROS1::GIT2*) identified by comprehensive genomic profiling (CGP), representing a previously unrecognized association. Introduction of entrectinib into the treatment algorithm of the patient was accompanied by significant clinical response.

The 19-month-old boy presented with visual disturbances as well as vertical nystagmus of the left eye and unstable gait. Magnetic resonance imaging (MRI) revealed multiple subcortical and periventricular white matter lesions also affecting the corpus callosum. One single, larger, homogenously contrast-enhancing, dural-based lesion was described in the right cerebellar hemisphere and another intradural extramedullary lesion in the level of the dens axis (Fig. 1a; Suppl. Fig. 1a). Outside the CNS, one contrast-enhancing lesion was found above the left lacrimal gland. On the control MRI performed 3 months later, unequivocal increase in the size of previously detected supratentorial, cerebellar, and spinal lesions was detected. Additionally, one novel contrast-enhancing soft-tissue lesion appeared behind the maxillary sinus (Suppl. Fig. 1b). Further investigations revealed no systemic involvement. Serological and immunological tests, electro-encephalography as well as cerebrospinal fluid cytology and flow cytometry were negative.

**Fig. 1:**
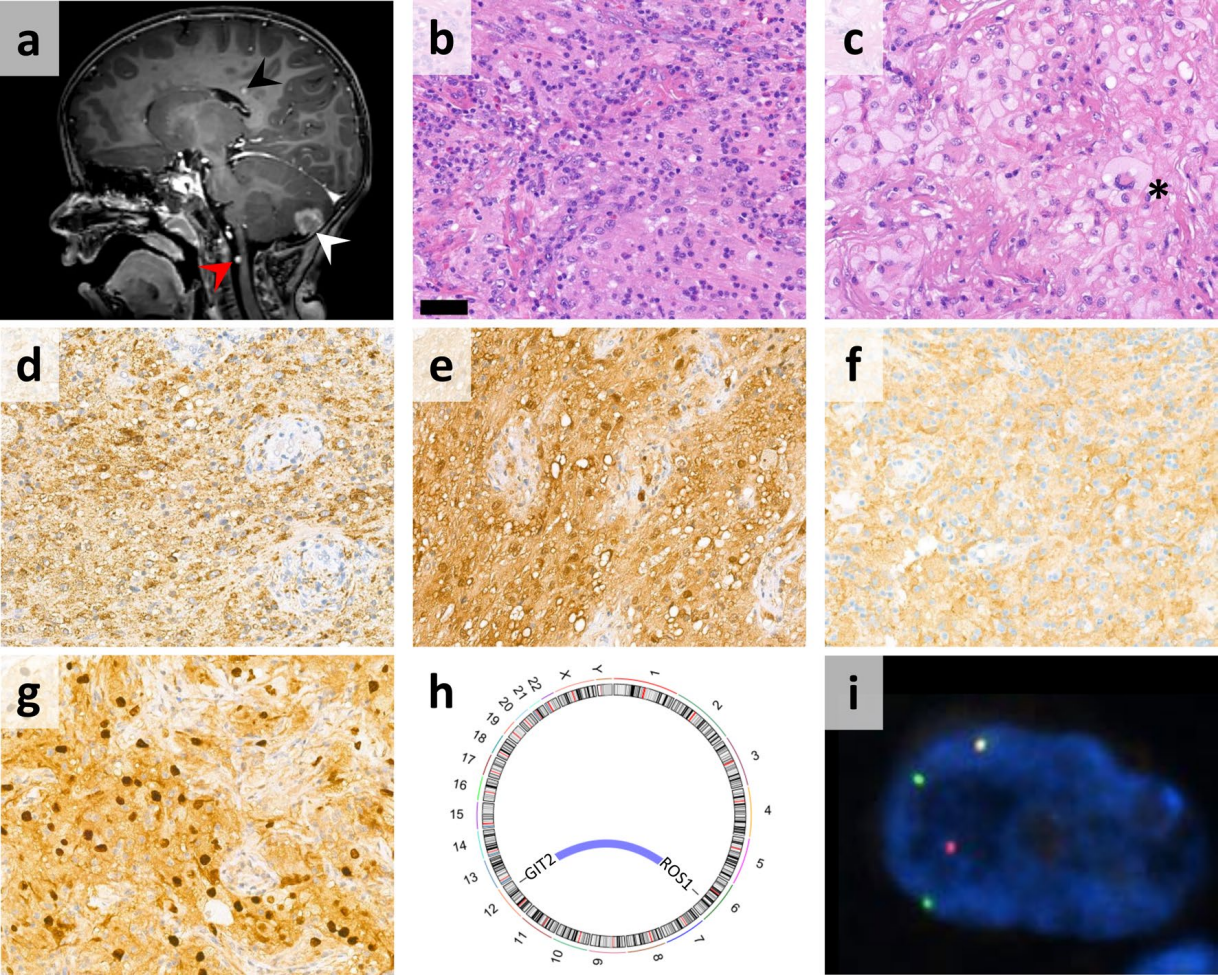
MRI (T1-weighted, contrast enhanced) (**a**) showed multiple white matter (black arrowhead) as well as single cerebellar (white arrowhead) and spinal extramedullary (red arrowhead) lesions. H&E-stained sections of the biopsy taken from the cerebellar mass showed neoplastic histiocytes with wide eosinophilic (**b**) or foamy, xanthomatous cytoplasm (**c**) and scattered Touton-type giant cells (asterix) (scale bar: 50 µm). Tumor cells showed positivity with CD68 (**d**), S100 (**e**), and ROS1 (**f**) immunohistochemical reactions and widespread strong nuclear positivity with cyclin D1 (**g**). Next-generation sequencing revealed *ROS1::GIT2* fusion (**h**) and FISH analysis using a break-apart probe confirmed the presence of *ROS1* rearrangement (**i**)

Biopsy was taken from the cerebellar lesion to explore the nature of the disease. Histology showed a dense infiltrate of neoplastic histiocytes in the cerebellar parenchyma and leptomeningeal surface. Cells contained wide eosinophilic cytoplasm in some areas, while foamy, xanthomatous cells and scattered Touton-type giant cells were observed in others. Nuclear atypia was mild to moderate and the mitotic activity was inconspicuous. A reactive infiltrate of lymphocytes and eosinophilic granulocytes as well as marked fibrosis were readily detectable (Fig. 1b, c). Tumor cells showed diffuse positivity with CD68 (Fig. 1d) and S100 (Fig. 1e) immunohistochemistry, while CD1a, langerin (CD207), and BRAF p.V600E reactions were negative. Focal strong positivity was detected with D5F3 ALK immunohistochemistry, but rearrangement affecting the *ALK* gene was not detected by fluorescent in situ hybridization (FISH). The final histological diagnosis was non-Langerhans cell histiocytosis with some morphological features of juvenile xanthogranuloma.

Next-generation sequencing (NGS) based CGP was performed using the Illumina TruSight Oncology 500 (TSO-500) assay (Suppl. table 1) as previously described [2]. This identified a novel fusion involving the *ROS1* (6q22.1; exon 36) and *GIT2* (12q24.11; exon 16) genes (Fig. 1h) which was validated using *ROS1* break-apart FISH probe (Fig. 1i) and direct Sanger sequencing of the fusion transcripts (Suppl. Fig. 2, Suppl. Table 2). Further targetable genetic alterations were not found (Suppl. Table 3). Ancillary immunohistochemical studies showed diffuse, weak to moderate cytoplasmic positivity with the D4D6 ROS1 antibody (Fig. 1f), and a widespread strong nuclear labeling with cyclin D1 (Fig. 1g).

Histology and immunophenotype of the tumor overlaps with Erdheim–Chester disease, juvenile xanthogranuloma and ALK-positive histiocytosis. Cyclin D1 overexpression has been reported in both Rosai–Dorfman disease [7] and ALK-positive histiocytosis and is related to both MAPK/ERK and PI3K/AKT/mTOR pathway activation [9]. Considering the similarity of ALK and ROS1 signal transduction, positivity of cyclin D1 in our *ROS1*-rearranged case is not surprising and underlies the utility of this marker in the diagnostics of histiocytoses.

Postoperative MRI showed no or mild changes in the size of known lesions, but novel sacral (S1-2) neuroforaminal foci appeared (Suppl. Fig. 1c). Bone scintigraphy and trephine biopsy were negative. The treatment was started with the DAL-HX 90 chemotherapeutic regimen including alternating “A” (etoposide, ifosfamide and methotrexate) and “B” (vinblastine, etoposide and methotrexate) blocks, completed with intrathecal triple therapy (methotrexate, prednisolone and cytarabine) and subcutaneous cladribine (see Suppl. Fig. 3). Following these, entrectinib treatment was chosen as a maintenance therapy based on the molecular report, with an off-label indication. Control MRI performed during the chemotherapy as well as 1 month and 4 months following the introduction of entrectinib showed radiologically stable disease. In the second month of entrectinib treatment, clear improvement of visual disturbances was clinically detectable, although a visual field defect of the right eye was suspected and the nystagmus was still present. Six months following the entrectinib treatment initiation, by the time of the preparation of this manuscript, the visual field defect was no longer evident, and the patient could show parts of tiny objects and imitate the examiner’s gesticulation.

ROS1 is a receptor tyrosine kinase with poorly characterized physiological function. However, fusions affecting this gene were described in several benign and malignant tumors including a subset of non-small-cell lung carcinomas (NSCLC) [11]. These rearrangements are characterized by diverse partner genes and breakpoints found between exon 32 and 36 of *ROS1* resulting in constitutive activity of the kinase domain [5]. *GIT2* encodes an ADP ribosylation factor (ARF) GTPase activating protein (GAP). *GIT2::BRAF* and *GIT2::PDGFRB* fusions were described in pilocytic astrocytoma [8] and chronic myeloproliferative disorders [12], respectively, with breakpoints in exon 12 of *GIT2*. Altered protein–protein interactions related to ankyrin and ARF-GAP domains of *GIT2* are the presumed mechanism of oncogene activation [12]. The *ROS1::GIT2* fusion identified in our patient has not been reported previously, but the breakpoints suggest mechanisms similar to the abovementioned ones.

Entrectinib is a small molecular tyrosine kinase inhibitor with efficient blood–brain barrier penetration and favorable side-effect profile. The compound was approved by the FDA and EMA for the treatment of adult patients with NSCLC harboring *ROS1* fusion [1]. The radiological stabilization and apparent clinical improvement in our case suggest the utility of this compound in *ROS1*-rearranged histiocytosis as well. To the best of our knowledge, we reported here the first case of successful disease control using entrectinib treatment in non-Langerhans cell histiocytosis with novel *ROS1::GIT2* fusion. Although this association needs to be confirmed in additional cases, our report clearly highlights the utility of CGP in histiocytic neoplasms.

## Supplementary Information

Below is the link to the electronic supplementary material.

Suppl. Fig. 1 MR images (T1-weighted, contrast-enhanced) showing the soft-tissue lesion detected behind the maxillary sinus (a), the spinal extramedullary lesion in the level of dens axis (b) and a lumbo-sacral (S1-2) neuroforaminal lesion (c) (white arrowheads)

Suppl. Fig. 2 Electropherogram of Sanger-sequencing which validated the novel ROS1::GIT2 fusion on cDNA level on the reverse strand

Suppl. Fig. 3 Timeline demonstrating the diagnostic procedures and therapeutic interventions during the clinical course of the reported case in months from the initial presentation (CNS: central nervous system, ETP: etoposide, IFO: ifosfamide, i.th.: intrathecal, i.v.: intravenous, MRI: magnetic resonance imaging, MTX: methotrexate, pred.: prednisolone, VBL: vinblastine)

Suppl. Table 1 List of genes analyzed by the Illumina TruSight Oncology 500 for small variants (single nucleotide variants and small insertions & deletions), fusions and copy number variations, respectively (source: https://www.illumina.com/products/by-type/clinical-research-products/trusight-oncology-500.html). Suppl. table 2 Genomic sequence of the detected novel ROS1::GIT2 fusion (*indicates the breakpoint) and sequences of the custom-designed primers applied for the validation of the fusion with Sanger-sequencing. Suppl. table 3 Relevant small variants, fusions and copy number variations detected by the Illumina TruSight Oncology 500 next-generations sequencing analysis in the reported case
