# Supplementary figures and images for: Novel actionable ROS1::GIT2 fusion in non-Langerhans cell histiocytosis with central nervous system involvement
Source: Acta Neuropathol. 2022 Nov 23;145(1):153–6. doi: 10.1007/s00401-022-02520-6 (PMC9807475; doi:10.1007/s00401-022-02520-6)

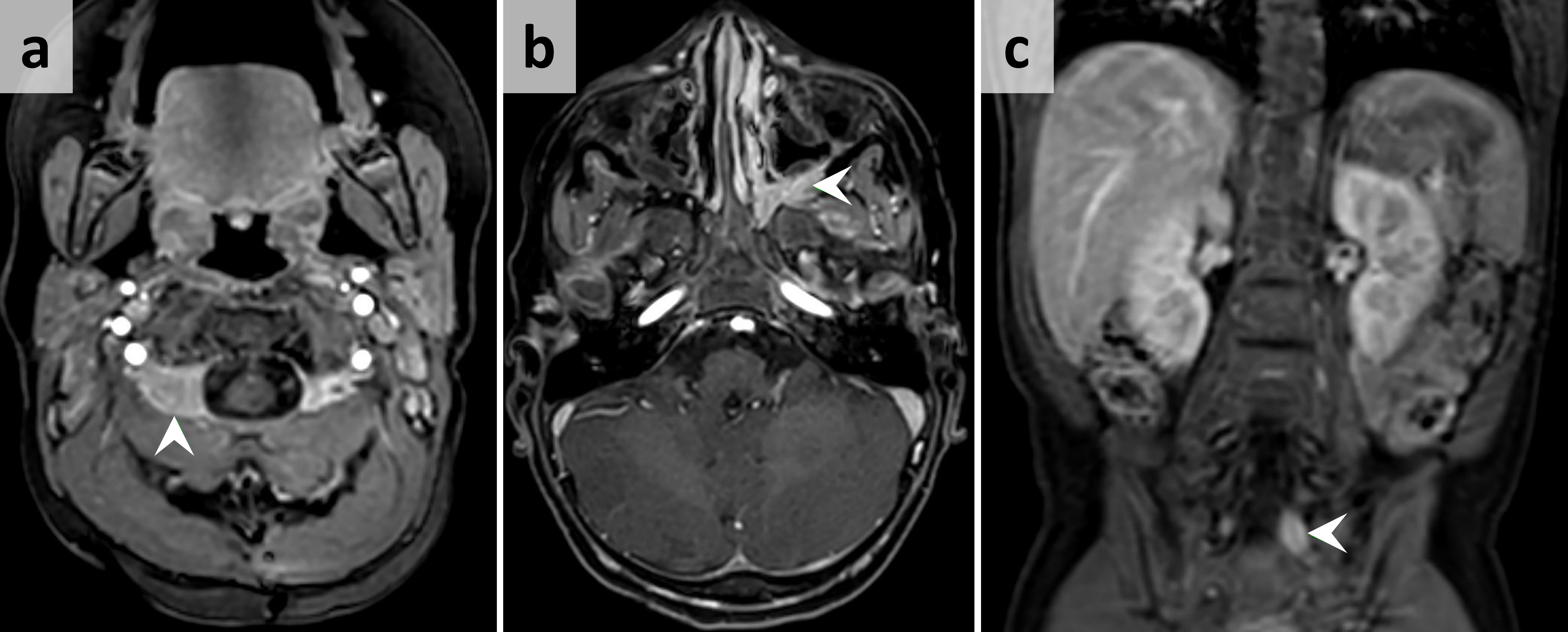

Supplement: Supplementary file 1 — Suppl. Fig. 1 MR images (T1-weighted, contrast-enhanced) showing the soft-tissue lesion detected behind the maxillary sinus (a), the spinal extramedullary lesion in the level of dens axis (b) and a lumbo-sacral (S1-2) neuroforaminal lesion (c) (white arrowheads) [file 401_2022_2520_MOESM1_ESM.tif]

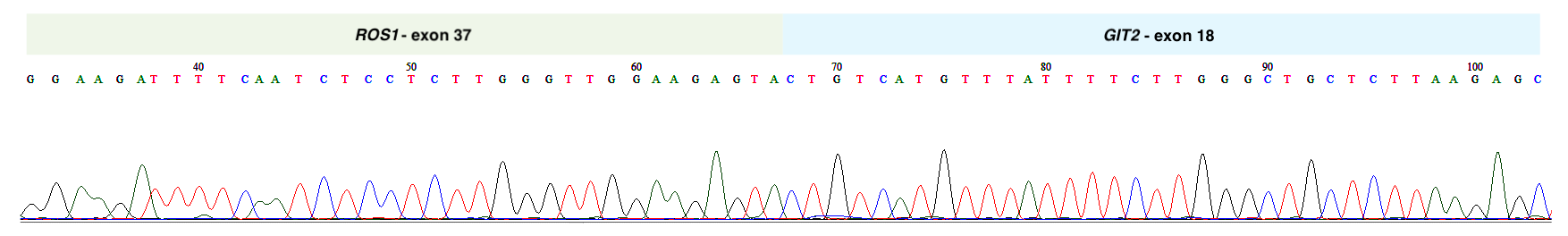

Supplement: Supplementary file 2 — Suppl. Fig. 2 Electropherogram of Sanger-sequencing which validated the novel ROS1::GIT2 fusion on cDNA level on the reverse strand [file 401_2022_2520_MOESM2_ESM.tiff]

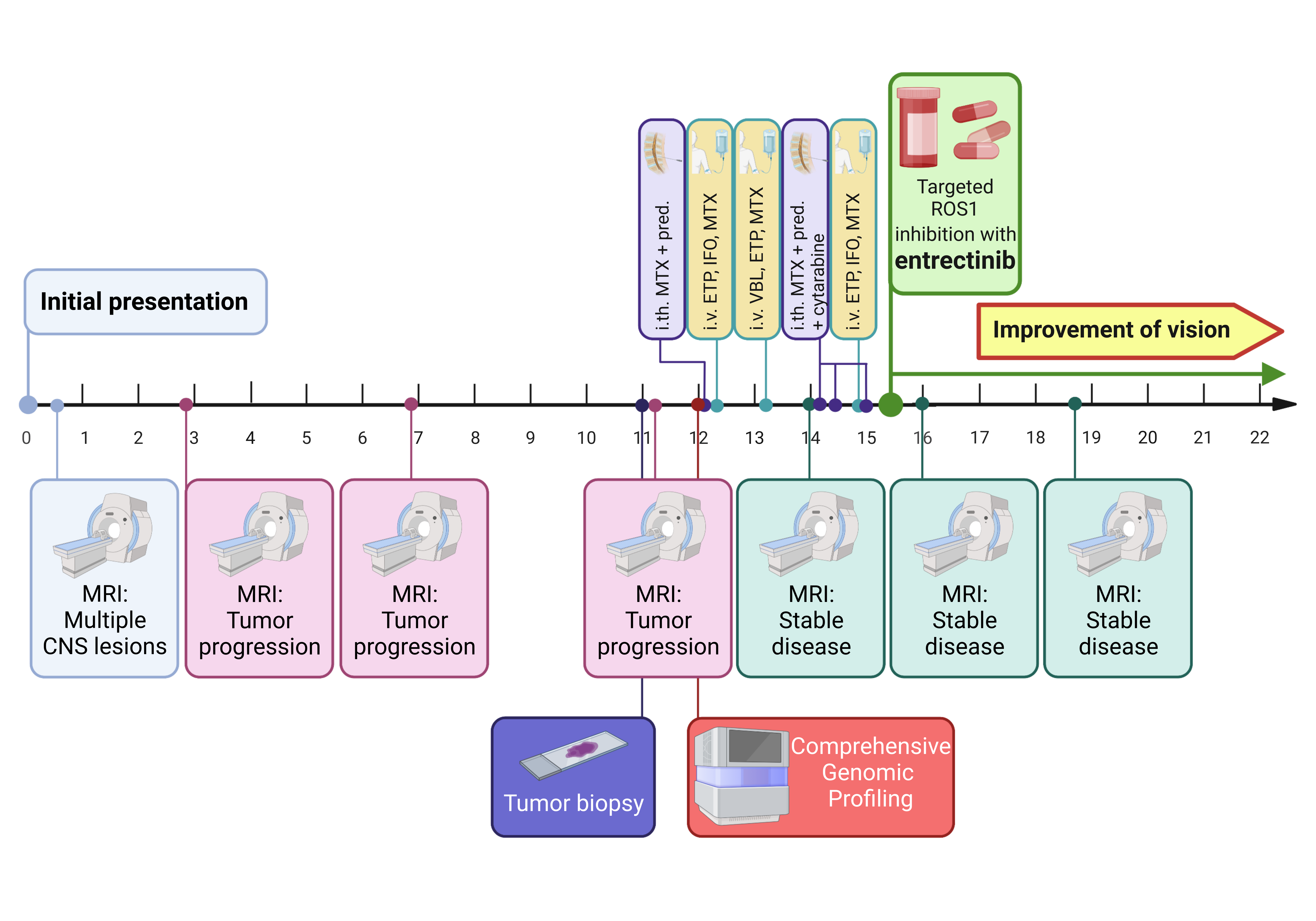

Supplement: Supplementary file 3 — Suppl. Fig. 3 Timeline demonstrating the diagnostic procedures and therapeutic interventions during the clinical course of the reported case in months from the initial presentation (CNS: central nervous system, ETP: etoposide, IFO: ifosfamide, i.th.: intrathecal, i.v.: intravenous, MRI: magnetic resonance imaging, MTX: methotrexate, pred.: prednisolone, VBL: vinblastine) [file 401_2022_2520_MOESM3_ESM.tif]
